# XPolaris: an R-package to retrieve United States soil data at 30-meter resolution

**DOI:** 10.1186/s13104-021-05729-y

**Published:** 2021-08-26

**Authors:** Luiz H. Moro Rosso, Andre F. de Borja Reis, Adrian A. Correndo, Ignacio A. Ciampitti

**Affiliations:** Department of Agronomy, Kansas State University, Manhattan, KS 66506 USA

**Keywords:** Programming, Agriculture, Soil properties, Maps, POLARIS

## Abstract

**Objectives:**

This data article aims to introduce the “XPolaris” R-package, designed to facilitate access to detailed soil data at any geographical location within the contiguous United States (CONUS). Without the need of advanced R-programming skills, XPolaris enables users to convert raster data from the POLARIS database into traditional spreadsheet format [i.e., Comma-Separated Values (CSV)] for further data analyses.

**Data description:**

The core of this publication is a code-tutorial envisioned to assist users in retrieving soil raster data within the CONUS. All data is sourced from the POLARIS database, a 30-m probabilistic map of soil series and different soil properties [Chaney et al. Geoderma 274:54, 2016, Chaney et al. Water Resour Res 55:2916, 2019]. POLARIS represents an optimization of the Soil Survey Geographic (SSURGO) database, circumventing issues of spatial disaggregation, harmonizing, and filling spatial gaps. POLARIS was constructed using a machine learning algorithm, the Disaggregation and Harmonisation of Soil Map Units Through Resampled Classification Trees (DSMART-HPC) [Odgers et al. Geoderma 214:91, 2014]. Although the data is easily accessible in a raster format, retrieving large amounts of data can be time-consuming or require advanced programming skills.

## Objective

The objective of this dataset [1] is to introduce the R-package “XPolaris”, a collection of functions for retrieving soil data from the POLARIS database [2, 3]. Although POLARIS raster images are easily accessible and a client API (Application Programming Interface) has been recently released [4], programming skills are necessary to retrieve large amounts of data. Therefore, the core functionalities of XPolaris will facilitate accessing soil data regardless of the number of geographical locations. Due to a large volume of data in each raster image, efficient coding is necessary to match the user need with a minimum download requirement. Examples of research publications taking advantage of soil information from the POLARIS database are presented below:

In [5], gridded soil data (soil organic matter, clay, silt, and sand at 0–15 cm) was obtained for 679 site-years across North America. The research aimed to predict corn yield using a machine learning algorithm (conditional random forests). About 50% of corn yield variability was explained by crop management and soil variables, with previous crop and soil organic matter as the most relevant features.

In [6], soil water variables (ksat, θ_saturated_, θ_resdiual_, and van Genuchten–Mualem parameters) from 95 US locations were used in the SWAP model for simulating crop evapotranspiration reduction (drought stress). The project aimed to predict soybean biological nitrogen fixation using linear model regularization (elastic net). This method identified soil and weather variables most strongly associated with nitrogen fixation (40% of evaluated features).

## Data description

Data files are deposited in the Harvard Dataverse repository “Retrieving POLARIS data using R-software” [1]. The RMarkdown file (*.rmd) (Data file 1 in Table 1) was generated using R version 4.0.3 (MacOS, 64-bit) and R-studio v1.4.1103. It intends to present XPolaris and its core functionalities. There is no limit on the amount of data retrieved by the user. However, the image download depends on internet connection and large objects can surpass the memory limit of the R environment and/or machine. The code chunks must be executed in the order they are presented in the RMarkdown file. Users can replace the location data with their own.

**Table 1:** Overview of data files/data sets

Label	Name of data file/dataset	File types (file extension)	Data repository and identifier (DOI or accession number)
Data file 1	XPolaris_code	RMarkdown file (.rmd)	Harvard Dataverse: 10.7910/DVN/DCZ0N3 [1]
Data file 2	XPolaris_tutorial	PDF file (.pdf)	Harvard Dataverse: 10.7910/DVN/DCZ0N3 [1]
Data file 3	XPolaris_documentation	PDF file (.pdf)	Harvard Dataverse: 10.7910/DVN/DCZ0N3 [1]
Dataset 1	exkansas	CSV file (.csv)	Harvard Dataverse: 10.7910/DVN/DCZ0N3 [1]

In the tutorial portable document file (*.pdf) (Data file 2 in Table 1) users are introduced to the input format (Sect. “Introduction” of the tutorial) and the three functions related to: (1) checking images from which location data must be retrieved (Sect. “Location areas”); (2) downloading raster images covering requested soil variables and depths (Sect. “Downloading images”); and (3) extracting the soil data from the images to generate a CSV output for further analyses (Sect. “Extracting soil data”). Details on the function arguments are included in another portable document file (*.pdf) (Data file 3 in Table 1).

The POLARIS database provides 13 soil variables (Data file 2 in Table 1) related to physical and chemical properties (e.g., soil organic matter, pH, clay, silt, sand, bulk density, ksat, etc.) at six different depth layers (0–5, 5–15, 15–30, 30–60, 60–100, and 100–200 cm) and a 30-m spatial resolution. Because the database was constructed from a probabilistic model [7], values are summarized by their mean, mode, median (p50), 5th (p5) and 95th (p95) percentiles. All POLARIS raster files use a geographic coordinate system (GCS) and the WGS84 datum.

The CSV file (Dataset 1 in Table 1) is an example of location input, containing three geographical coordinates in Kansas for which soil data will be retrieved and the R functions will be tested. The example data also comes with the XPolaris package [8]. XPolaris facilitates code implementation by exempting users from writing extensive functions. In addition, the package was tested across different operating systems, being released in CRAN [9].

## Limitations

- The local machine must have available disk space to store the raster images.
- Visualization functions are not included for the retrieved soil data.
- Currently, soil data cannot be summarized within spatial polygons.
- Soil data output is not directly compatible with crop simulation models (e.g., APSIM, DSSAT).

## Data Availability

The data described in this Data note can be freely and openly accessed on Harvard Dataverse: https://doi.org/10.7910/DVN/DCZ0N3 [[Bibr CR1]]. Please see Table 1 for details about the data.

## Abbreviations

SWAP: Soil Water Atmosphere and Plant
ksat: Saturated hydraulic conductivity
θ_saturated_: Saturated soil water content
θ_resdiual_: Residual water content
DSMART: Disaggregation and Harmonisation of Soil Map Units Through Resampled Classification Trees
SSURGO: Soil Survey Geographic database
CSV: Comma-Separated Values
DSSAT: Decision Support System for Agrotechnology Transfer
